# The Mid-Pleistocene Transition: a delayed response to an increasing positive feedback?

**DOI:** 10.1007/s00382-022-06544-2

**Published:** 2022-11-04

**Authors:** J. D. Shackleton, M. J. Follows, P. J. Thomas, A. W. Omta

**Affiliations:** 1 Department of Earth, Atmospheric, and Planetary Sciences, Massachusetts Institute of Technology, Cambridge, MA 02139 USA; 2 Department of Mathematics, Applied Mathematics and Statistics, Case Western Reserve University, Cleveland, OH 44106 USA; 3 Department of Earth, Environmental, and Planetary Sciences, Case Western Reserve University, Cleveland, OH 44106 USA

**Keywords:** Glacial cycles, Mid-Pleistocene Transition, Bifurcation, Carbon cycle, Feedbacks

## Abstract

Glacial–interglacial cycles constitute large natural variations in Earth’s climate. The Mid-Pleistocene Transition (MPT) marks a shift of the dominant periodicity of these climate cycles from $\sim 40$ to $\sim 100$ kyr. Recently, it has been suggested that this shift resulted from a gradual increase in the internal period (or equivalently, a decrease in the natural frequency) of the system. As a result, the system would then have locked to ever higher multiples of the external forcing period. We find that the internal period is sensitive to the strength of positive feedbacks in the climate system. Using a carbon cycle model in which feedbacks between calcifier populations and ocean alkalinity mediate atmospheric CO$_2,$ we simulate stepwise periodicity changes similar to the MPT through such a mechanism. Due to the internal dynamics of the system, the periodicity shift occurs up to millions of years after the change in the feedback strength is imposed. This suggests that the cause for the MPT may have occurred a significant time before the observed periodicity shift.

## Introduction

**Fig. 1:**
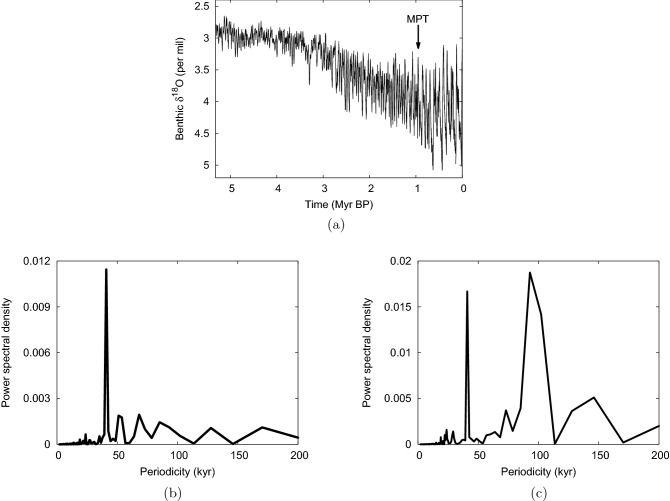
**a** Benthic foraminifera $\delta ^{18}$O, a proxy for global ice volume and ocean temperature, over the past 3 Myr (Lisiecki and Raymo 2005); reproduced from Omta et al. (2016). Note that time goes forward to the right. **b** Periodogram of $\delta ^{18}$O for the time window between 2 and 1 Myr ago; note the dominant peak at $\sim 40$ kyr. **c** Periodogram of $\delta ^{18}$O for the window between 1 Myr ago and the present; note the dominant peak at $\sim 100$ kyr

Ever since Ice Ages were discovered in the nineteenth century, there has been discussion about their causes. Initially, explanations focused on the role of variations in the Earth’s orbit (Croll 1875; Milankovitch 1941). However, the exact relationship between the orbital forcing and global climate is still not entirely clear and certainly not straightforward (Crucifix 2012; Paillard 2015; Berger et al. 2016). One particularly intriguing feature is the Mid-Pleistocene Transition (MPT). At about 1 Myr ago, the dominant periodicity of the glacial–interglacial cycles lengthened from around 40 kyr to around 100 kyr (Fig. 1). Both before and after the MPT, the strongest orbital climate forcings have been precession, with a $\sim 20$-kyr period, and obliquity, with a $\sim 40$-kyr period (Berger and Loutre 1991; Laskar et al. 2004; Huybers 2006; Berends et al. 2021). How could such a change in the qualitative behavior of the system have occurred without a clear change in the orbital forcing?

Several hypotheses have focused on the Northern hemisphere ice sheets. Clark and Pollard (1998) suggested that the MPT was caused by gradual soil erosion underneath the Laurentide ice sheet. Originally, this ice sheet was mostly on top of soft soils, but erosion gradually put more of the ice sheet in direct contact with hard bedrock. This solid foundation would then have allowed the ice sheet to grow thicker and oscillate more slowly. Raymo et al. (2006) and Huybers and Tziperman (2008) suggested that the MPT was related to the ice sheets extending ever further toward the Equator. Bintanja and van de Wal (2008) pointed out that the thicker North American ice sheets of the Late Pleistocene could more easily survive insolation maxima and would therefore melt only every few orbital cycles. Related to this hypothesis is the idea that Earth’s climate has three steady states: “interglacial”, “mild glacial”, and “full glacial” (Paillard 1998). It has been suggested that the Earth was oscillating between the “mild glacial” and “interglacial” states before the MPT, whereas it subsequently started oscillating between the “full glacial” and “interglacial” states (Ditlevsen 2009; Ashwin and Ditlevsen 2015). This mechanism would then have led to cycles with a larger amplitude and a longer period after the MPT.

Glacial–interglacial cycles are characterized not only by large climatic variations but also by shifts in the global carbon cycle. Both before and after the MPT, atmospheric CO$_2$ appears to have varied approximately in step with ice volume (Lüthi et al. 2008; Hönisch et al. 2009; Higgins et al. 2015; Chalk et al. 2017; Dyez et al. 2018). One of the first hypotheses to explain the MPT involving the carbon cycle was formulated by Saltzman and Maasch (1991). According to this hypothesis, a gradual decrease in the average atmospheric CO$_2$ concentration throughout the Pleistocene culminated in the climate system becoming unstable, which then led to the large-amplitude, long-period, asymmetric cycles of the Late Pleistocene. Recently, Quinn et al. (2018) applied a quasi-periodic insolation forcing to the Saltzman and Maasch (1991) model. In this system, the duration and amplitude of the modeled glacial–interglacial cycles increased seemingly spontaneously around the time of the MPT. The calcifier-alkalinity model (Omta et al. 2013) describes the glacial–interglacial cycles as an oscillation of the ocean carbon cycle and coupled atmospheric CO$_2$ through changes in ocean Ca$^{2+}$ and associated alkalinity. Omta et al. (2016) showed that different periodicities emerge when a periodic forcing is applied to this model. Periodicity shifts were then found to occur due to either noise or the quasi-periodic nature of the astronomical forcing. According to the Glaciator model, the MPT occurred due to the expansion of C4 plants, which increased the efficiency of photosynthesis and led to a crossing of a Hopf bifurcation (Arnaut and Ibáñez 2020). Furthermore, it has been suggested that the MPT was the result of an increased carbon storage capacity in the ocean due to a reorganization of the overturning circulation (Lear et al. 2016; Kender et al. 2018; Farmer et al. 2019).

Finally, some hypothesized explanations do not invoke specific physical, chemical, or biological mechanisms but rather the subtleties of nonlinear dynamics. One example is the Huybers (2009) model that exhibits changes in the dominant periodicity of the cycles without a change in the system parameters or the forcing. A key assumption behind this model is that there exists a memory in the climate system, which makes ice sheets melt faster after a cold glacial period than after a relatively mild glacial. Another example is a hypothesis suggested by Rial et al. (2013): the 100-kyr cycles would have emerged due to frequency modulation by the 413-kyr eccentricity component. Furthermore, Mitsui et al. (2015) suggested that a decrease in the natural frequency shifted the system from a 40-kyr mode locked state to either quasi-periodic cycles or a strange non-chaotic attractor.

At this point, it appears difficult to distinguish between these many different hypotheses. Therefore, some recent studies have attempted to identify features of the MPT as targets for models to reproduce. One such feature is an approximately linear relationship between the length and amplitude of each cycle (Omta et al. 2016). Another feature is the stepwise increase in the average duration from 40 to 80 to 120 kyr (Nyman and Ditlevsen 2019). Nyman and Ditlevsen (2019) demonstrated that such stepwise changes can be caused by a gradual increase in the internal period (or equivalently, a decrease in the natural frequency) in combination with frequency locking to an external (Milankovitch) forcing. In Nyman and Ditlevsen (2019), the internal period of the modeled cycles was predetermined by an imposed threshold. By contrast, Verbitsky et al. (2018) showed that longer cycles can emerge from an increase in the strengths of positive feedbacks in a system. Could positive feedbacks play a role in the ramping with frequency locking (RFL) mechanism identified by Nyman and Ditlevsen (2019)? If so, how do such feedbacks affect RFL? The calcifier-alkalinity model (Omta et al. 2013) *allows us to investigate these questions, because it* generates sawtooth cycles without a threshold and exhibits frequency locking to external periodic forcing (Omta et al. 2016). Since this model is also simple and versatile, we think it is a particularly suitable tool for our purpose.

In Sect. 2, we briefly restate the model formulation and discuss its physical interpretation. Furthermore, we discuss the model experiments and the tools and criteria we use to assess the results. In Sect. 3, we first demonstrate that the internal period of the system increases with increasing positive feedback strength. Subsequently, we investigate the system’s response to changes in the feedback strength. We show that a change in cycle duration can occur a significant time after the feedback change. In Sect. 4, we discuss the robustness and implications of our results. We think that the key mechanism likely applies to other dynamical models for sawtooth cycles as well, since it simply relies on the system having a finite response time to changes in the external forcing. We conclude with a summary of the key findings in Sect. 5.

## Methods

In Sect. 2.1, we describe the variables and parameters of the calcifier-alkalinity model. Although this model focuses on only one component of the Earth system (ocean alkalinity and its control on atmospheric CO$_2$), it provides a simple and tractable tool to study the interplay between frequency locking of sawtooth cycles and positive feedbacks. Previous research on this model is briefly highlighted and an extension is made to the model for our current research. Subsequently, we focus on the technical implementation of the model, as well as the concepts and tools we use to analyze the results (Sect. 2.2).

### Model formulation

The calcifier-alkalinity model (Omta et al. 2013) centers on ocean alkalinity (*A*) and its impact on atmospheric CO$_2.$ Alkalinity is defined as the net positive charge difference between “conservative” ions (e.g., Na$^+,$ Ca$^{2+},$ Cl$^-$) that act as neither bases nor acids under oceanic conditions (Chester 2000) or, equivalently, as the net surplus of bases over acids (Dickson 1981). Being an acid, CO$_2$ reacts with bases and therefore, a higher ocean alkalinity is associated with a larger storage capacity for carbon. In practice, the ocean alkalinity cycle largely revolves around the input and output of Ca$^{2+}$ ions. Ca$^{2+}$ is continuously transported into the oceans as a consequence of rock weathering on the continents. Incorporation of CaCO$_3$ into the shells of calcifying organisms and subsequent sedimentation of these shells removes Ca$^{2+}$ ions from the ocean.

According to the model, the weathering input creates a gradual increase of alkalinity from an interglacial toward a glacial maximum, leading to uptake of carbon from the atmosphere into the ocean. The glacial–interglacial transitions are then associated with spikes in calcifying organisms (*C*), which bury CaCO$_3$ and create sharp drops in alkalinity, leading to outgassing of carbon from the ocean into the atmosphere. Thus, the model generates sawtooth oscillations in alkalinity, corresponding to the reverse sawtooth cycles in CO$_2$ observed in ice cores (Petit et al. 1999; Augustin et al. 2004; Lüthi et al. 2008). In its simplest form, the model formulation is as follows:

1a $$\begin{aligned} \frac{dA}{dt}&= I - kAC \end{aligned}$$

1b $$\begin{aligned} \frac{dC}{dt}&= kAC - MC \end{aligned}$$

with weathering input of alkalinity at rate *I*,  the calcifier population growing at rate *kA*,  and calcite sedimentation occurring at rate *M*. We follow the transformation performed in Omta et al. (2016), $P \equiv \ln \left( C \right) ,$ for purposes of numerical stability:

2a $$\begin{aligned} \frac{dA}{dt}= I - kA\exp \left( P \right) \end{aligned}$$

2b $$\begin{aligned} \frac{dP}{dt}= kA - M. \end{aligned}$$

The periodic forcing acts on the reaction rate *k* (as in Omta et al. 2013, 2016), because observations from various locations at different latitudes have shown Milankovitch cycles in CaCO$_3$ accumulation (Beaufort et al. 1997; Herbert 1997):

3 $$\begin{aligned} k = k_0\left( 1 + \alpha \cos \left( \frac{2\pi t}{T}\right) \right) . \end{aligned}$$

**Table 1:** List of parameters with values and units reproduced from Omta et al. (2016) as our parameter values do not change from the previous study

Parameter	Units	Meaning	Value
*I*	$\text{mol}\,\text{eq}\,\text{m}^{-3}\,\text{year}^{-1}$	Alkalinity input	$4\times 10^{-6}$
$k_0$	$\left( \text{mol}\,\text{eq}\right) ^{-1}\,\text{m}^3\,\text{year}^{-1}$	Reaction rate	0.05
*M*	$\text{year}^{-1}$	Sedimentation rate	0.1
$\alpha$	–	Periodic forcing amp.	Variable

We differ slightly from Omta et al. (2013, 2016), which generally used a (precessional) forcing with a period of 20 kyr. As recent studies have suggested that obliquity is the primary forcing of the glacial–interglacial cycles (Bajo et al. 2020; O’Neill and Broccoli 2021), we now set our forcing period *T* to 40 kyr unless otherwise specified. The other parameter values are kept the same as in Omta et al. (2016) as shown in Table 1. The model sustains sawtooth cycles with periods equal to multiples of the forcing period. Omta et al. (2016) demonstrated that the system moves between different multiples of the forcing period when subject to noise, imposed on the parameter *k*.

Verbitsky et al. (2018) showed that changes in the strengths of positive feedbacks provide an alternative mechanism to affect the periodicity of modeled sawtooth cycles. To probe this mechanism, we add a positive feedback to the weathering parameter *I*. This implies that the alkalinity input increases during times of high alkalinity and decreases at low alkalinity, a type of feedback that was discussed but not implemented in Omta et al. (2016). As we discuss in Sect. 4.1, a potential mechanism underlying such a feedback would be enhanced weathering input from exposed continental shelves during glacial times and decreased weathering input during interglacials. Since there are limits to the feedback and the continental shelves are finite, we impose bounds on the feedback term in our model. For this purpose, we propose the use of a simple sigmoid function ($\arctan$) that tends towards constant values at low and high alkalinity: $I=I_0 \left( 1 + \gamma S\left( A\right) \right) ,$ with $S\left( A\right) = \frac{2}{\pi }\tan ^{-1}\left( \frac{\pi \left( A - A_0\right) }{2 z_0}\right) ,$ with average alkalinity $A_0=2.0$ mM eq and scaling parameter $z_0=0.1$ mM eq. Hence, Eq. (2) become:

4a $$\begin{aligned} \frac{dA}{dt}= I_0\left( 1 + \gamma S\left( A\right) \right) - kA\exp \left( P \right) \end{aligned}$$

4b $$\begin{aligned} \frac{dP}{dt}= kA - M. \end{aligned}$$

The parameter $\gamma$ sets the relative change in the weathering input parameter *I*: $I \approx I_0 \left( 1+\gamma \right)$ for $A \gg A_0$ and $I \approx I_0 \left( 1-\gamma \right)$ for $A \ll A_0.$ Typically we consider feedback strengths in the range $0\le \gamma \lesssim 0.12$ which corresponds to deviations from average alkalinity input, *I*,  up to $12\%.$

### Model implementation and analysis

Simulations are performed in Julia version 1.5.3 using a Differential Equations package (Rackauckas and Nie 2017). We use the KenCarp58 solver for our system with a tolerance of $10^{-16}.$ Sample code is available on GitHub.^1^

To analyze the simulation results, we use concepts from dynamical systems theory; a thorough introduction can be found in Guckenheimer and Holmes (1985). Of particular relevance here are stable equilibria, limit cycles, and the supercritical Hopf bifurcation. A stable equilibrium is a constant value that the system tends toward asymptotically as time approaches infinity. Another possibility is that the system tends toward a stable limit cycle, which is an isolated, closed periodic orbit. Such an orbit indicates the existence of an oscillation that is continuously sustained without external forcing. As a system parameter is shifted, the equilibrium may lose stability, giving way to a stable limit cycle through a supercritical Hopf bifurcation.

**Fig. 2:**
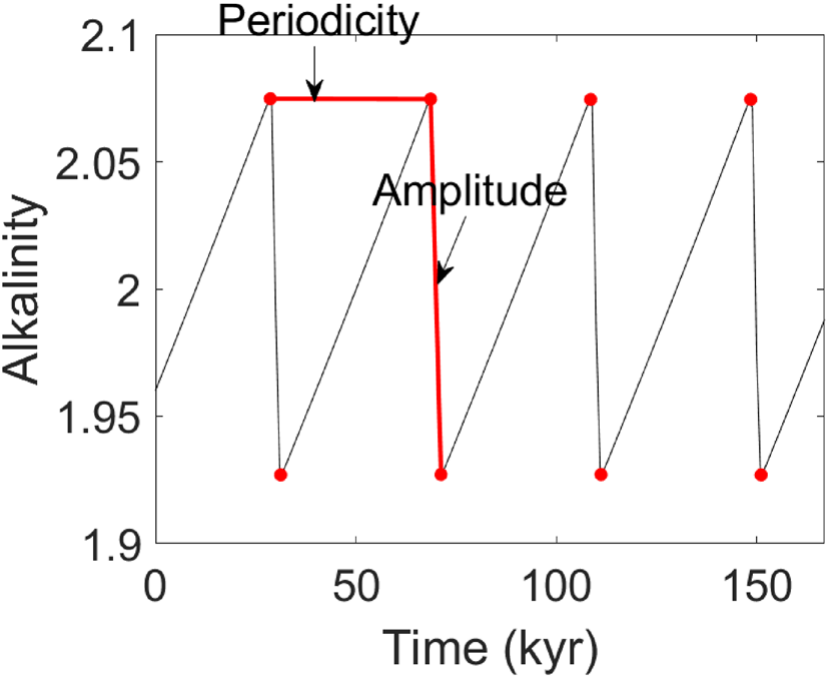
Minimum and maximum peaks are used to calculate the amplitude and periodicity of sawtooth cycles

As in Omta et al. (2016), we define periodicity as the time between successive local maxima of the alkalinity, and amplitude as the difference in alkalinity between a maximum and minimum, as seen in Fig. 2. In addition, we use concepts and notation as defined in Nyman and Ditlevsen (2019). The internal period, ${\mathbf{T}}_{\mathbf{0}},$ is the periodicity that the solution would tend toward asymptotically if the forcing amplitude were zero. We use ${\mathbf{T}}_{\mathbf{0}}$ as a metric, since it depends on the model parameters and succinctly describes the behavior of the unforced solution. The average duration, ${\mathbf{D}},$ is the periodicity that the system tends toward asymptotically when the forcing amplitude is non-zero. Following Nyman and Ditlevsen (2019), we plot the average duration as a function of the internal period. This plot has a staircase-like structure that is known as a Devil’s Staircase. The internal period and average duration are calculated by running our simulation for 100 million years and averaging the final 50 cycles. Although it generally only takes around 20 million years for the periodicity to converge close to a steady value, we choose to simulate for 100 million years as a reasonable limit for long-term behavior. However, the results are not very sensitive to the chosen simulation time (as long as the solution approximately represents the simulation’s long-term behavior).

The main algorithm used for finding the periodicity and amplitude is a peak-finding algorithm from the Python scipy signal package find_peaks.^2^ The function finds the maximum and minimum peaks of the sawtooth cycle which easily allows the calculation of both periodicity and amplitude. A depiction of this method can be seen in Fig. 2. The red dots are the peaks found by the algorithm and the lines connecting the peaks show the calculation of amplitude and periodicity.

## Results

Using a model in which glacial–interglacial transitions were controlled by a set threshold, Nyman and Ditlevsen (2019) produced stepwise increases in the periodicity of the cycles through the ramping with frequency locking (RFL) mechanism. The ramping component of this mechanism refers to a gradual increase in the internal period of the system. Furthermore, a periodically forced system may exhibit cycles with an average duration that is close to its internal period and is a multiple of the forcing period. This is the frequency locking phenomenon, which effectively modifies the average duration to “fit” the external forcing. For a system with a constant external forcing, increasing the internal period by changing one of the system parameters can then lead to jumps from one locked period multiple to another. Is this RFL mechanism also found in the calcifier-alkalinity model, in which the glacial–interglacial transitions are not set by a threshold but rather emerge from its internal dynamics?

To investigate this question, we must first find a suitable parameter for changing the internal period of our system. Verbitsky et al. (2018) indicated that increasing positive feedbacks increases the internal period of the system. Our system includes a positive feedback through the exposure of continental shelves encapsulated by the parameter $\gamma$ (discussed in more detail in Sect. 4.1). An investigation of the impact of $\gamma$ on the internal period and average duration indeed shows that it is a viable parameter to use for RFL (Sect. 3.1). We then explore the impact of an increase in $\gamma$ during a simulation (Sect. 3.2).

### Impact of feedback strength on internal period and average duration

**Fig. 3:**
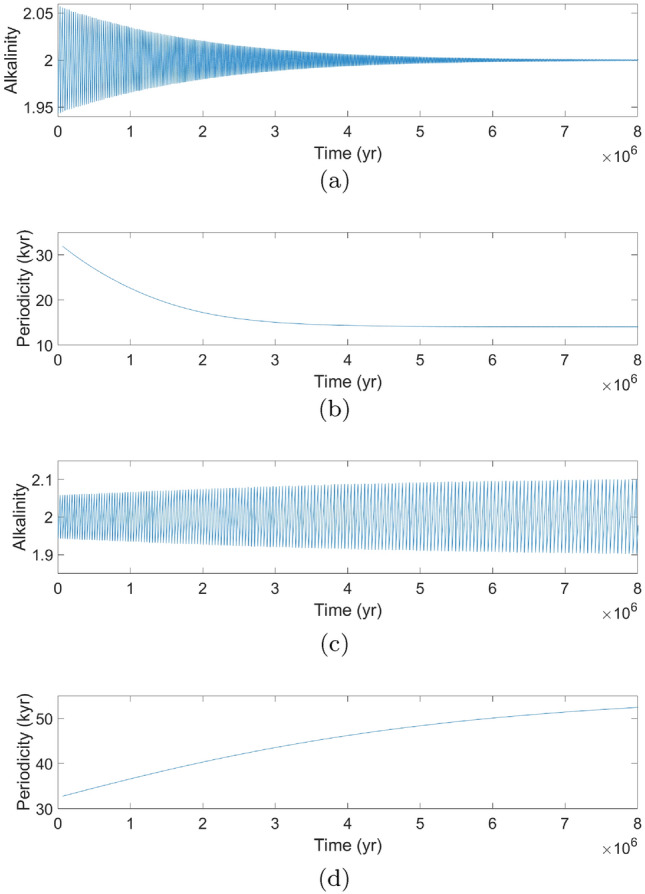
**a** Shows the solution of our model for feedback strength $\gamma = 0.02$ and external forcing amplitude $\alpha = 0.$ These parameter values lead to oscillations decaying to a fixed value of alkalinity since there is no external forcing and the value of $\gamma$ is before the Hopf Bifurcation. **b** Shows **a** from the view of periodicity as it decays to a fixed value of periodicity of about 14 kyr. **c** and **d** Show the solution for $\gamma = 0.07.$ Stable oscillations exist since $\gamma$ is beyond the bifurcation and periodicity converges to about 55 kyr

**Fig. 4:**
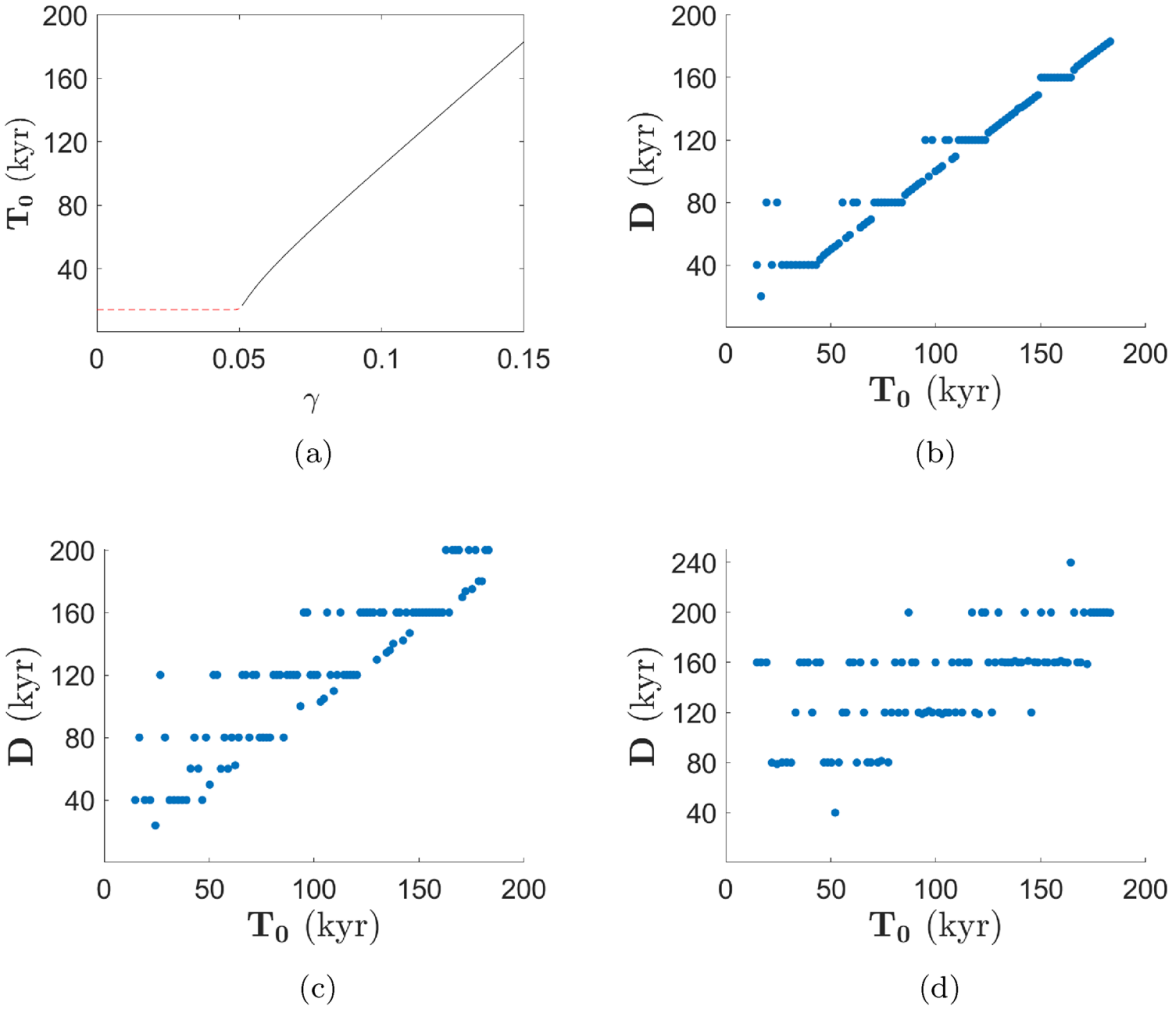
**a** Shows internal period, ${\textbf {T}}_0,$ as a function of feedback strength, $\gamma .$ The cycles are not self-sustaining before the supercritical Hopf bifurcation at $\gamma = 0.05$ (see stability calculation in Appendix 1) and are denoted with a red-dashed line. The internal period roughly increases linearly with $\gamma$ beyond the bifurcation. **b**–**d** Show the average duration, ${{\textbf {D}}},$ dependent on internal period for values of external forcing strengths of $\alpha = 0.001,$ 0.003,  and 0.008 respectively. The graphs only consider internal period and average duration beyond the bifurcation

**Fig. 5:**
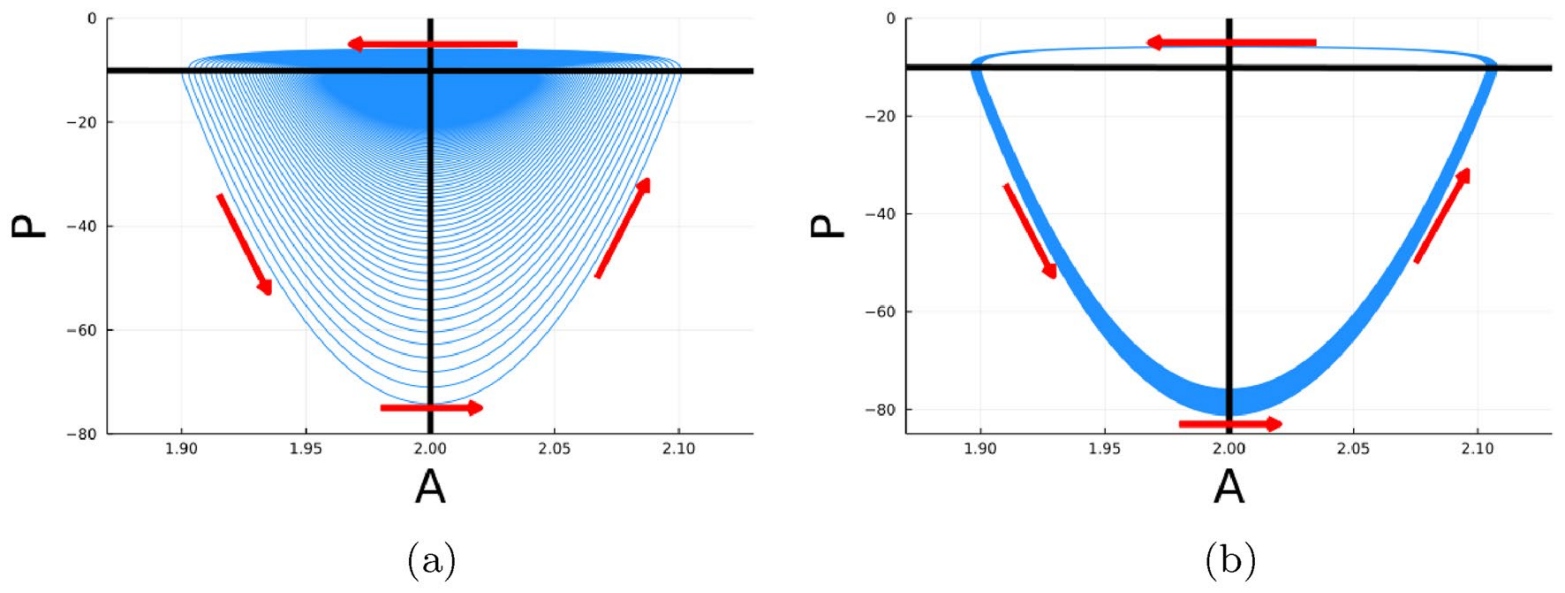
Phase plane plots of model simulations for $\gamma = 0.02$ (**a**) and $\gamma = 0.07$ (**b**), with the log calcifiers (*P*) graphed with respect to alkalinity (*A*). The horizontal line is the *A*-nullcline, or where the derivative of *A* with respect to time is 0, and the vertical line is the *P*-nullcline. Arrows indicate the direction the solution flows in the phase plane; generally, the top arrow specifies the fast change from a glacial to interglacial period while the other arrows show the slow movement from an interglacial into a glacial period. In **a**, the solution spirals to the equilibrium or, in other words, the intersection of the two nullclines. Beyond the Hopf bifurcation, the solution tends towards a fixed cycle such as in **b**

Without astronomical forcing and without sufficiently strong feedback, the system exhibits decaying oscillations with an internal period (inverse of natural frequency) of 14 kyr (Fig. 3a). When the feedback is included, the unforced system undergoes a supercritical Hopf bifurcation at a feedback strength $\gamma = 0.05$ (see stability calculation in Appendix 1). The behavior around $\gamma = 0.05$ is typical of a system undergoing a supercritical Hopf bifurcation. That is, the model exhibits asymptotic oscillations with a small amplitude and a sinusoidal shapes immediately beyond the bifurcation. Further beyond the supercritical Hopf bifurcation, the amplitude of the oscillations increases and obtain a progressively more nonlinear (sawtooth) character. Figure 3c is a solution to the calcifier-alkalinity model with feedback strength $\gamma = 0.07,$ which results in a stable oscillation. The periodicity, in Fig. 3d, converges to a single value of about 55 kyr, which can also be read from the internal period graph in Fig. 4a. This latter figure indicates the asymptotic periodicity of our system in the absence of external forcing ($\alpha = 0$) as a function of $\gamma .$ Before the Hopf bifurcation ($\gamma < 0.05$), alkalinity spirals toward the steady state with decreasing amplitude but a fixed internal period which negligibly changes dependent on the value of $\gamma$ (red-dashed line in Fig. 4a). This spiral to equilibrium is shown from a phase plane perspective in Fig. 5a for $\gamma = 0.02.$ Beyond the Hopf bifurcation ($\gamma > 0.05$), the system displays asymptotically stable sawtooth cycles with an internal period that increases roughly linearly with increasing $\gamma$ (black line in Fig. 4a). The trend towards a stable sawtooth cycle is seen in the phase plane in Fig. 5b for $\gamma = 0.07.$ The internal period reaches the largest periodicity seen in the ice-age cycles, 120 kyr, at a feedback strength of about 0.12. This value of feedback strength is reasonable since, indeed, it is plausible for alkalinity input to deviate from its mean value by about $10\%.$

The average duration is found using the same method as the internal period; namely, running the simulation for 100 million years and averaging the last 50 cycles. The plots of average duration as a function of internal period in Fig. 4b–d are created by finding the average duration as a function of $\gamma$ and using Fig. 4a to find the internal period for each value of $\gamma .$ To focus on the main feature of the staircase, we exclude values of internal period and average duration before the bifurcation at $\gamma =0.05.$

The periodicity begins to lock noticeably onto multiples of the forcing period when the amplitude of the external forcing reaches $\alpha = 0.001$ (Fig. 4b). The larger regions of locking in Fig. 4c for $\alpha = 0.003$ demonstrate the impact of increasing external forcing strength. However, we find that the main structure of the Devil’s Staircase is lost for $\alpha > 0.008.$ This phenomenon can be explained by the fact that the system becomes chaotic at $\alpha \approx 0.008$ (Omta et al. 2016) and more sensitive to initial conditions. Despite the influence of this phenomenon, frequency locking does, indeed, occur. The external forcing influences the average duration to favor multiples of the forcing period. Figure 4c shows a strong impact from the external forcing while also maintaining staircase-like structure. Therefore, this value of external forcing, $\alpha = 0.003,$ will be used for further study on dynamic jumps between the different steps of the staircase.

### Feedback strength shifts

**Fig. 6:**
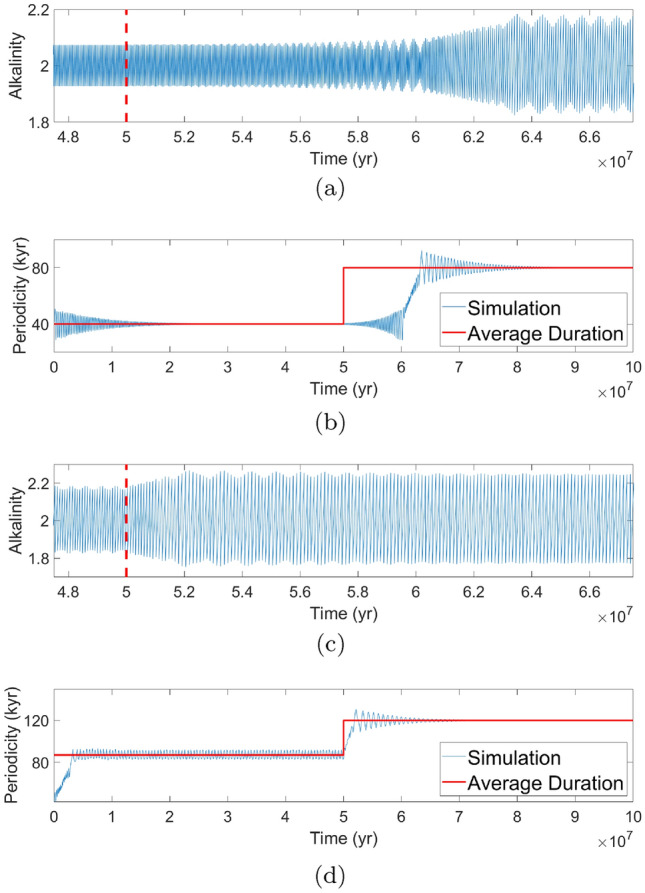
**a** and **c** are two different simulations with a sudden change in the feedback strength parameter $\gamma$ occurring $5\times 10^7$ years after the start, as highlighted with the dashed red line. **a** and **c** Show a movement from $\gamma = 0.06$ to $\gamma = 0.09$ and from $\gamma = 0.09$ to $\gamma = 0.12$ respectively, both with $\alpha = 0.003.$ The perspective of periodicity versus time is shown in **b** for $\gamma = 0.06$ to $\gamma = 0.09$ and **d** for $\gamma = 0.09$ to $\gamma = 0.12.$ **b** Demonstrates an exponentially decreasing envelope of periodicity to 40 kyr; after the change of parameter, there is an exponentially increasing envelope until the system becomes unstable and transitions to an exponentially decreasing envelope converging to 80-kyr periodicity. **d** On the other hand, shows a periodicity which oscillates around a value of about 87 kyr before jumping to an exponentially decreasing envelope converging to a 120-kyr periodicity

Nyman and Ditlevsen (2019) proposed RFL as a mechanism to explain the MPT. We have identified the feedback strength parameter $\gamma$ to be a prime candidate to produce RFL within our model. As we varied $\gamma ,$ the average duration moved steadily from 40 to 120 kyr with locking observed at multiples of forcing. In contrast with Nyman and Ditlevsen (2019), we now change the internal period abruptly by setting $\gamma (t)$ as a piecewise function: $\gamma (t) = \gamma _1$ before a transition time $t_c$ and $\gamma (t) = \gamma _2$ after this point in time. Although such an abrupt change is likely not realistic, it allows us to observe the transient behavior of the model after a change in $\gamma$ in a clear manner. We set $t_c = 5\times 10^7$ years after the start of the simulation to reduce the impact from transient movements at the beginning of the simulation. A particular focus is on $\gamma$ values of 0.06,  0.09,  and 0.12,  as these correspond to average durations of 40,  80,  and 120 kyr. After settling into a periodic orbit, corresponding to $\gamma = 0.06$ for Fig. 6a and $\gamma = 0.09$ for Fig. 6c (both with $\alpha = 0.003$), $\gamma$ changes to 0.09 and 0.12 respectively. The system becomes unstable and the periodicity oscillates with increasing amplitude until moving to the next periodicity multiple of the forcing period. The red-dashed lines in Fig. 6a, c indicate the time at which the jump of $\gamma$ occurs. Figure 6b, d show the same simulations as Fig. 6a, c but from the perspective of periodicity versus time, with the solid red lines indicating the average duration. Average duration is defined as the asymptotic periodicity of the system, which means that the average periodicity of the solution tends toward the red lines as it settles from transient effects.

Upon the sudden change in feedback strength, we noticed two different behaviors. As in Fig. 6b, the periodicity decays with some oscillating variation to a fixed value of 40 kyr, which is a multiple of the period of forcing. Alkalinity remains oscillating at this periodicity until it is disturbed by the change in $\gamma .$ The periodicity responds with growing oscillations. There appears a relatively short intermittent phase where periodicity is moving between the two multiples until it begins decaying to the 80-kyr periodicity multiple of forcing. Figure 6d shows a different behavior as the periodicity keeps oscillating around 87 kyr. The oscillation cannot decay to a fixed periodicity since the 87-kyr periodicity is not a multiple of the forcing period. This behavior is dependent on the initial conditions: for the same parameters and different initial conditions, the periodicity decays to a value of 80 kyr. Since the system is not exactly locked to 80 kyr in Fig. 6d, the periodicity moves much more quickly towards 120 kyr after the change in $\gamma .$ The time scale for Fig. 6b was around 10 Myr while for Fig. 6d the time scale was around 1 Myr.

**Fig. 7:**
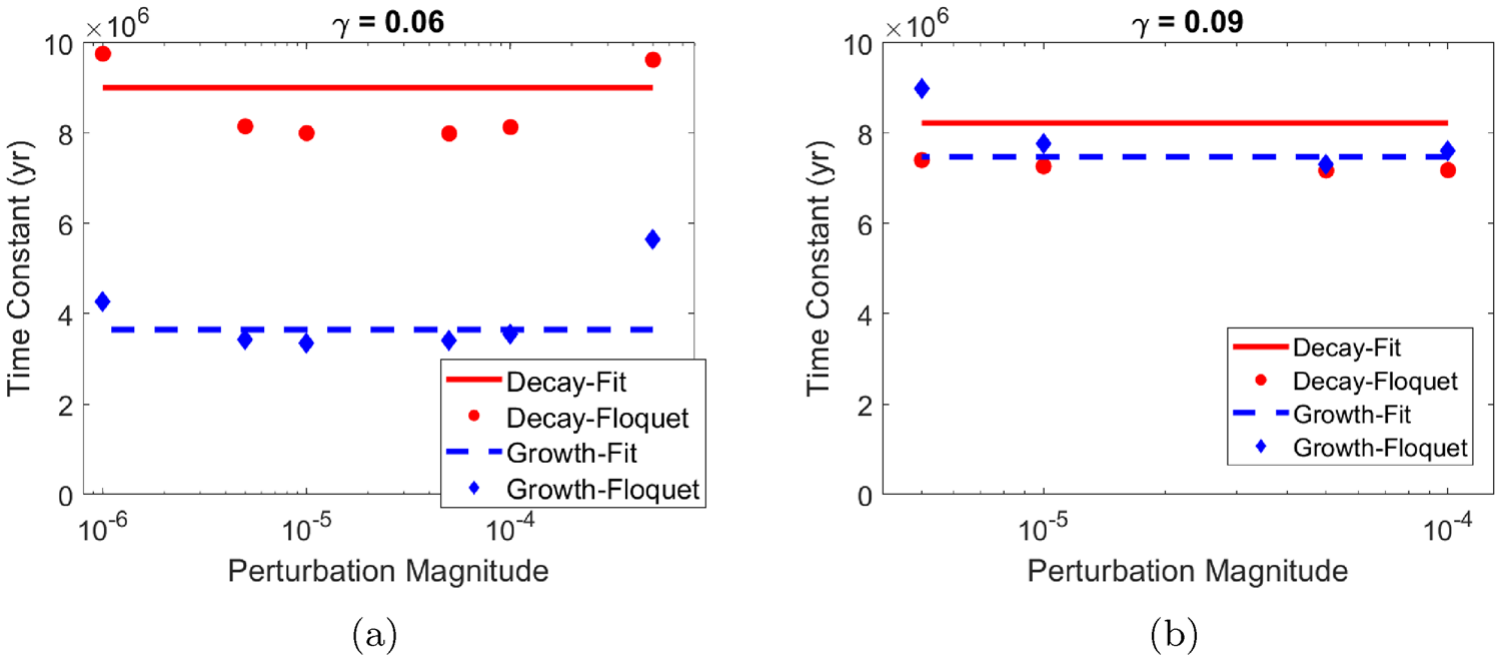
The calculated time constants for both exponential decay and growth are shown for both $\gamma = 0.06$ corresponding to a 40-kyr periodicity in **a** and $\gamma = 0.09$ corresponding to a 80-kyr periodicity in **b**. By fitting, the decay time constant for a 40-kyr periodicity is found to be 9 Myr while the growth time constant is 3.6 Myr. For an 80-kyr periodicity, the decay time constant is 8.2 Myr and the growth time constant is similarly 7.5 Myr. Calculations by Floquet analysis confirm the values found by fitting as there is general agreement between the two methods on the range of the time constant

What is the dynamical mechanism behind the delayed system response? One possibility is that after the shift in $\gamma ,$ the original oscillation still exists as an unstable limit cycle. As a result, the movement away from it may initially occur very slowly. Only once the distance from the original oscillation becomes sufficiently large, it loses its influence and the system starts to move away from it more rapidly. If this explanation is correct, then the initial rate at which the system moves toward (or away from) a fixed periodicity calculated by Floquet analysis should correspond with the rate obtained from a fit to the envelope of the cycles (for details, see Appendix 2). Results of these two approaches are summarized in Fig. 7a for a 40-kyr periodicity and Fig. 7b for an 80-kyr periodicity (with exact frequency locking). While the two approaches do not give identical answers, they do agree to a considerable degree. The system decays toward a 40-kyr periodicity with a time constant of about 9 Myr and grows away with a time constant of about 3.5 Myr after the change in $\gamma .$ For the 80-kyr oscillation, both the decay and growth time constants are around 8 Myr.

**Fig. 8:**
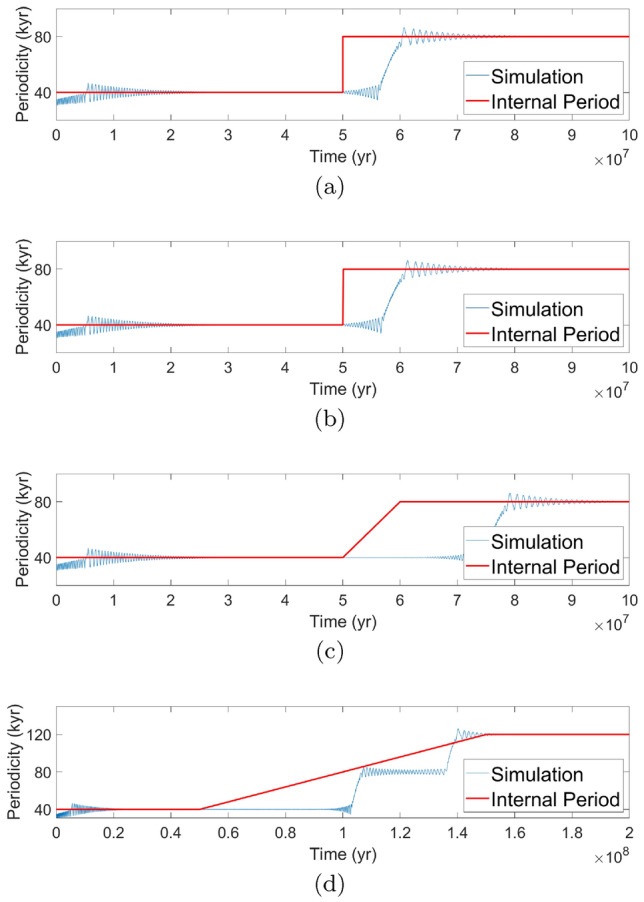
Simulations are performed for various continuous changes of the parameter $\gamma .$ **a** is a reference simulation with an instantaneous change from 0.06 to 0.09. **b** Linearly varies $\gamma$ from 0.06 to 0.09 over $10^5$ years whereas **c** increases $\gamma$ over $10^7$ years. An increase in $\gamma$ over $10^8$ years is performed in **d** from 0.06 to 0.12 to demonstrate a noticeable change in behavior. Note that we assume a perfectly linear relation between $\gamma$ and internal period in the creation of these plots, whereas Fig. 4a shows a non-exact linear relation

In our investigation of the impact from a dynamically changing value of $\gamma$ during a simulation, we chose an instantaneous change of $\gamma$ rather than a continuous linear increase. We did so to keep the analysis simple as, in fact, the analysis does not change significantly if we allow continuous rather than instantaneous change. In simulations where $\gamma$ is instead linearly increased from $\gamma _1$ to $\gamma _2$ over a total time of $\Delta t,$ the response does not noticeably change, as in Fig. 8b, until $\Delta t$ reaches the order of $10^7$ years shown in Fig. 8c. For changes of $\gamma$ from 0.06 to 0.09, 0.09 to 0.12, and 0.06 to 0.12, the increase in the total delay is of the order of the number of years during which the change in $\gamma$ takes place. The system does not appear to respond to the changing $\gamma$ until it reaches a certain threshold that would induce the jump in periodicity multiple. As indicated by Fig. 4a, c, these thresholds will be close to $\gamma$ values of 0.09 for 80-kyr cycles and 0.12 for 120-kyr cycles, and so would not be crossed until the continuous change is nearly completed. The only exception in which a noticeable difference in behavior is seen is for a very long (on the order of $10^8$ years) increase of $\gamma$ from 0.06 to 0.12. The system will respond, with an expected delay, by increasing periodicity to 80 kyr until jumping to a periodicity of 120 kyr. If the change of $\gamma$ is too quick (less than $10^8$ years) than it will skip the middle step and only jump to a periodicity of 120 kyr from a 40-kyr periodicity. The behavior in Fig. 8d is similar to two instant jumps of $\gamma$ from 0.06 to 0.09 and then 0.09 to 0.12 some time after the first change. Thus, the simulation is consistent with our understanding of the system primarily reacting to $\gamma$ crossing threshold values.

## Discussion

The MPT represents a change in the periodicity of the glacial–interglacial cycles around 1 Myr ago. Although the dominant spectral peak shifted from $\sim 40$ kyr to $\sim 100$ kyr, it has been suggested that the lengths of individual cycles moved from a $\sim 40$-kyr to an $\sim 80$-kyr cycle and then to $\sim 120$-kyr (Huybers and Wunsch 2005; Daruka and Ditlevsen 2016; Omta et al. 2016; Nyman and Ditlevsen 2019). Ramping with frequency locking (RFL) is a mechanism proposed to explain the MPT consistent with this concept (Nyman and Ditlevsen 2019). The ramping refers to an increase in the internal period of the system, which then locks to multiples of the periodic forcing. We have demonstrated that RFL can be achieved in the calcifier-alkalinity model (Omta et al. 2013, 2016) through the inclusion of a positive feedback term. In Sect. 4.1, we discuss predictions made by the calcifier-alkalinity model. First, we focus on predictions that are tied to the physical interpretation of the model. Subsequently, we take a broader Earth system perspective. In particular, we discuss how positive feedbacks could play a key role in RFL regardless of the specific physical mechanism underlying the glacial–interglacial cycles. In Sect. 4.2, we compare RFL in our model with the Nyman and Ditlevsen (2019) study. In Sect. 4.3, we focus on the impacts of obliquity versus precession forcing according to our simulation results.

### Model predictions

As discussed in Sect. 2.1, the calcifier-alkalinity model describes sawtooth cycles in ocean alkalinity, corresponding with reverse sawtooth cycles in atmospheric CO$_2$ (as observed in ice cores). Although there is no proxy for alkalinity, the model makes the following potentially testable predictions:

1. the occurrence of CaCO$_3$ accumulation maxima at glacial–interglacial transitions. For this prediction, there exists a significant body of observational evidence (Flores et al. 2003; Jaccard et al. 2005, 2013; Brunelle et al. 2010; Rickaby et al. 2010).
2. a linear proportionality between the length and amplitude of glacial–interglacial cycles. We tested this prediction using $\delta ^{18}$O data from the past 3 Myr (Lisiecki and Raymo 2005) and found good agreement between model and data with regard to the overall trend (see Fig. 8 in Omta et al. 2016).
3. a correlation between the length/amplitude of glacial cycles and the magnitude of deglacial CaCO$_3$ accumulation spikes. Although this prediction is the most difficult to test, there is proxy evidence for increasing deglacial productivity maxima across the MPT (Schefuß et al. 2005; Hasenfratz et al. 2019).

Within the context of the calcifier-alkalinity model, the feedback term implies that there is an enhanced alkalinity input during glacial times and a decreased alkalinity input during interglacials. A potential mechanism behind such variations in alkalinity input is the exposure of continental shelves during glacial periods, which increases carbonate weathering (Gibbs and Kump 1994; Jones et al. 2002). Furthermore, the smaller extent of coral reefs during glacial times could decrease the output of alkalinity (Berger 1982). Even so, decreased silicate weathering during cold periods (Walker et al. 1981; White et al. 1999) may provide a competing negative effect. For an overall positive feedback, the larger exposed continental shelves and smaller coral reefs would need to outweigh the decreased silicate weathering during glacial times. An increasing value of $\gamma$ then suggests that the glacial–interglacial changes in exposed continental shelf extent would have increased over time. This may have occurred as a result of increases in the peak glacial ice volume, which occurred both in the early Pleistocene and during the MPT (see the lower envelope of the curve in Fig. 1).

That said, our conclusions about the potential role of a positive feedback in generating the MPT are likely not dependent on the specific feedback mechanism. For example, Verbitsky et al. (2018) also found that a positive feedback could lengthen the cycles over time. The Verbitsky et al. (2018) model did not include alkalinity: its three variables were the glaciated area, ice-sheet basal temperature, and characteristic temperature of outside-of-glacier climate. They encapsulated the combined strengths of the feedbacks in their model into a single variable *V* and increased this parameter to produce an increase in the periodicity similar to the MPT. The reason why a positive feedback induces longer cycles in two such different models is that it brings the system further out of equilibrium. This increases the amplitude of the cycles, which in turn implies an increase in the average duration of the cycles due to the sawtooth geometry (Omta et al. 2016). Thus, any increasing positive feedback could be of interest for future investigations of the MPT and the RFL mechanism. For example, the surface area of the Northern hemisphere ice sheets during peak glacial time appears to have increased strongly in the early Pleistocene (Batchelor et al. 2019). This, in turn, may have led to a strong increase in the ice-albedo feedback and larger glacial–interglacial variations in the terrestrial carbon cycle. Although these specific changes would have occurred a significant time before the MPT, our simulation results suggest that they could be relevant due to a delayed response of the cycles.

### Comparison with Nyman and Ditlevsen (2019)

Similar to Nyman and Ditlevsen (2019), we modeled shifts in the average duration of glacial–interglacial cycles through RFL. Nevertheless, there are salient differences between Nyman and Ditlevsen (2019) and our current study, in terms of the model formulation, the mechanisms involved, and the simulation results. In the model used by Nyman and Ditlevsen (2019), a deglaciation occurs whenever ice volume hit a certain threshold (as in, e.g., Wunsch 2003; Ashkenazy and Tziperman 2004; Paillard and Parrenin 2004; Huybers 2007; Imbrie et al. 2011). Thus, the overall sawtooth geometry and the amplitude and duration of the modeled cycles are set by this imposed boundary. In contrast, our model generates the entire sawtooth through its internal dynamics. To prevent the solution from spinning to infinity and consistent with the finite size of continental shelves, we included bounds on the feedback in the form of a saturating sigmoidal function. However, these bounds do not determine either the sawtooth geometry or the amplitude and average duration of the simulated cycles. In fact, we simulated periodicity changes without changing $z_0,$ the scaling parameter that determines where the sigmoid saturates, instead varying the feedback strength parameter $\gamma .$ Essentially, the mechanism behind the internal period increases in our study is that the system is kicked further out of equilibrium by a stronger feedback, whereas the mechanism in Nyman and Ditlevsen (2019) is an increase in the threshold. Although we are not able to rule out either mechanism, we think that the large number of candidate processes (see, e.g., Weinans et al. 2021 and references therein) makes changes in positive feedbacks a plausible mechanism.

With regard to the results, a key difference between our simulations and Nyman and Ditlevsen (2019) is the transient response. An increase in feedback strength has a less direct impact on the cycles than an imposed threshold increase. As a result, we found a delay between the parameter change and the change in periodicity of $\sim 1$–10 Myr. Although these values depend on the specifics of the model setup, the mechanism giving rise to the delay seems robust. That is, the dynamics need some finite time to adjust after a change in the underlying system. In our view, there is an interesting analogy with delayed Hopf bifurcations that have been described for certain slow-fast dynamical systems (Izhikevich 2000; Han et al. 2016): the system exhibits a change in its behavior a significant time after the causal event. In other words, the change in the Earth system that caused the MPT may have occurred a considerable time before the MPT. We believe that this finding has immediate implications for investigation of the MPT, in particular because many observational studies have focused on events during the MPT itself (Elderfield et al. 2012; Pena and Goldstein 2014; Kender et al. 2018; Farmer et al. 2019; Hasenfratz et al. 2019; Worne et al. 2020; Yehudai et al. 2021).

### Role of obliquity versus precession

There has been a long-running debate about the relative importance of precession and obliquity in forcing the glacial–interglacial cycles. According to classical Milankovitch (1941) theory, summer insolation at 65$^{\circ }$ N controls the size of Northern Hemisphere ice sheets. This leads to the prediction that precession should be the dominant forcing (Raymo and Nisancioglu 2003). Even so, it was recognized early on that the precession, obliquity, and eccentricity components of insolation all have high coherences with benthic foraminifera $\delta ^{18}$O (Hays et al. 1976; Imbrie et al. 1993). Based on the Rayleigh test for phase directionality, Huybers and Wunsch (2005) suggested that glacial–interglacial cycles are paced by obliquity. Furthermore, Omta et al. (2016) found that the durations of individual glacial–interglacial cycles cluster around multiples of the $\sim 40$ kyr obliquity period. A recent study also argued for obliquity as the dominant forcing, even though the timing of glacial–interglacial transitions was found to correlate with the phases of both precession and obliquity (Bajo et al. 2020). In our simulations, the possibility of a ‘double jump’ (a change of periodicity twice the external period) implies that a 20-kyr forcing could give rise to a 40-, 80-, 120-kyr succession of dominant glacial cycle periodicities. Physically, the change to 20 kyr would signify that the dominant Milankovitch cycle is precession rather than obliquity. With our model, roughly the same response is obtained with a 20- and a 40-kyr forcing (see Appendix 3). This result holds even for a mixture of the two forcings. In summary, the key factor is not the period of the external forcing, but rather the size of the change in the internal period.

## Conclusion

The Nyman and Ditlevsen (2019) model showed periodicity changes similar to the MPT due to an increase in the internal period of the system, whereas Verbitsky et al. (2018) demonstrated an increase in the period as a result of a strengthening positive feedback. We have combined these two mechanisms by including a positive feedback in the calcifier-alkalinity model (Omta et al. 2013, 2016). This feedback then allows the model to achieve periodicity jumps through RFL. Although we induce an instantaneous change of the asymptotic internal period through jumps of the feedback strength parameter, the actual period of our system does not change instantaneously. Essentially, the system needs time to adjust and find its new internal period.

The possibility of a delay between a parameter change and the resulting periodicity shift suggests that the fundamental change in the Earth system giving rise to the MPT may have occurred a significant time before the observed periodicity shift. Therefore, we believe that investigations into the cause of the MPT should not be limited to co-occurring changes in the system. Rather, changes that occurred a significant time before the MPT may also be considered. In particular, the strong increase in peak Northern hemisphere ice volume and surface area during the early Pleistocene could be of interest.

## Figures and Tables

**Fig. 9:**
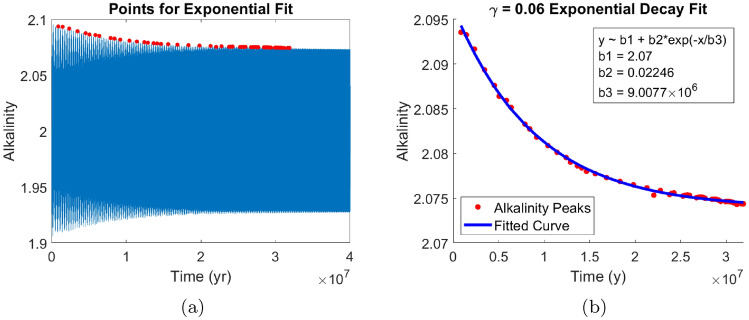
An example plot is shown where a fit is placed on alkalinity exponentially decreasing to a fixed amplitude and a fixed periodicity. The points chosen are shown in **a** which shows a simulation with $\gamma = 0.06$ asymptotically approaching a 40-kyr periodicity. The fit and calculated parameters are shown in **b** where the time constant for this example is found to be 9 Myr

**Fig. 10:**
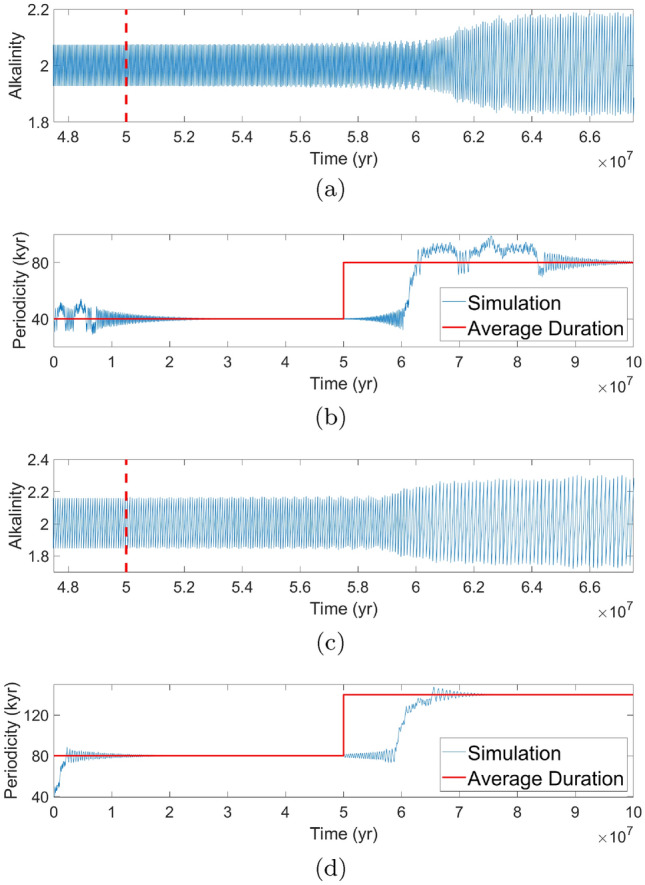
Simulations with a sudden change in $\gamma$ (as in Fig. 6) using a forcing with a 20-kyr (precession) rather than a 40-kyr (obliquity) period. As before, **a** and **c** show a movement from $\gamma = 0.06$ to $\gamma = 0.09$ and from $\gamma = 0.09$ to $\gamma = 0.12$ both with $\alpha = 0.003.$ Periodicity versus time is shown in **b** and **d** for the $\gamma = 0.06$ to $\gamma = 0.09$ and $\gamma = 0.09$ to $\gamma = 0.12$ transitions

## Data Availability

This is a theoretical/modeling study that did not generate any data. Model code is available through https://github.com/GlacialCycles/Example-Code.

## Appendix 1: Stability calculation

We use a method common for analyzing differential equations known as a stability calculation to gain an intuitive understanding of the impact of changing feedback strength on the system. Although finding general analytic solutions is usually impossible for non-linear differential equations, a first-order approximation of the model behavior close to the steady state can be found by evaluating the Jacobian matrix at steady state ($J^*$). At the (supercritical) Hopf bifurcation, a stable equilibrium becomes unstable and a limit cycle oscillation emerges. In a two-variable system, this corresponds with Tr$(J^*)=0,$ $\det (J^*)>0,$ and $d\left( \text{Tr}(J^*)\right) /d\gamma > 0$ at the equilibrium (van Voorn and Kooi 2017). Thus, we determine the regions of parameter space where the equilibrium is stable. Note that calculations are for the solution without forcing, i.e., $\alpha = 0.$

The equilibrium point is found when both derivatives are zero or when $\left( A^*, \exp \left( P^*\right) \right) = \left( \frac{M}{k}, \frac{I}{M}\right) .$ Linearizing around the equilibrium, the Jacobian of our system is

$$J^*=\left[\begin{array}{ll} -k\exp \left( P^*\right) &{} -kA^*\exp \left( P^*\right) \\ k &{} 0 \end{array}\right].$$

Substituting the previously found values of $\exp \left( P^*\right)$ and $A^*,$ we obtain

$$J^*=\left[\begin{array}{ll} -\frac{kI}{M} &{} -I \\ k &{} 0 \end{array}\right].$$

The trace is always negative, thus without forcing the solution spirals towards the fixed point while decreasing in amplitude until reaching a steady state. However, the feedback term for the parameter *I* changes the Jacobian to be:

$$\left[\begin{array}{ll} \frac{I\gamma }{z_0\left( 1 + \left( A^* - A_0\right) ^2\right) }-k\exp \left( P^*\right) & -kA^*\exp \left( P^*\right) \\ k & 0 \end{array}\right].$$

Again, substituting the values for $\exp \left( P^*\right)$ and $A^*$ and assuming that $A_0 = A^*,$ the Jacobian becomes

$$\left[\begin{array}{ll} \frac{I\gamma }{z_0}-\frac{kI}{M} &{} -I \\ k &{} 0 \end{array}\right].$$

The trace is positive for $\gamma > \frac{kz_0}{M}$ ($=0.05$ for standard parameter values), and the derivative of the trace with respect to $\gamma$ is $I/z_0$ which is positive for our standard parameter values as well. In other words, the Hopf bifurcation occurs as $\gamma$ increases past 0.05. The Hopf bifurcation was found to be supercritical analytically using the generalized equation for the first Lyapunov coefficient shown in Kuznetsov (2006) with further detail found in Section 3.5 of Kuznetsov (2004). The first Lyapunov coefficient was calculated to be $-1.188\times 10^{-8}$; the negative sign of the coefficient indicates that the Hopf bifurcation is supercritical. Solutions increase in amplitude near the equilibrium point and eventually settle on a stable limit cycle.

## Appendix 2: Time constant calculations

Our system generally converges to a fixed periodicity that is a multiple of the forcing period. This convergence is marked by an exponentially decreasing periodicity to the fixed value. Upon becoming unstable by changing $\gamma ,$ the system has an exponentially increasing periodicity tending away from the fixed value. Our goal is to understand and quantify this behavior.

We rely on an approach inspired by Floquet analysis. Consider our system with $\gamma = 0.06$ converging to 40-kyr periodicity as an example. If we look at the system in phase space, with coordinates (*A*, *P*),  then there is a point ${\mathbf{p}}$ such that after 40 kyr the system returns to point ${\mathbf{p}}.$ If the system were at point ${\mathbf{x}}_{\mathbf{0}},$ then it would move to point ${\mathbf{x}}_{\mathbf{1}}$ after 40 kyr where ${\mathbf{x}}_{\mathbf{0}} \ne {\mathbf{x}}_{\mathbf{1}}.$ Sufficiently close to ${\mathbf{p}},$ we can relate points ${\mathbf{x}}_{\mathbf{0}}$ and ${\mathbf{x}}_{\mathbf{1}}$ with the Monodromy matrix $\text{M}.$ That is,

5 $$\begin{aligned} {\mathbf{x}}_{\mathbf{1}} - {\mathbf{p}} = \text{M}\left( {\mathbf{x}}_{\mathbf{0}} - {\mathbf{p}}\right) + {\mathcal {O}}\left( |{\mathbf{x}}_{\mathbf{0}}-{\mathbf{p}}|^2\right) \end{aligned}$$

where $|{\mathbf{x}}_{\mathbf{0}}-{\mathbf{p}}|,$ the Euclidean norm of the displacement between vectors ${\mathbf{x}}_{\mathbf{0}}$ and ${\mathbf{p}},$ is taken to be small. (Here $f(u)={\mathcal {O}}(u^2)$ represents any function *f*(*u*) such that $\left| f(u)/u^2\right|$ is bounded by a finite constant in the limit as $u\rightarrow 0.$) We can calculate the matrix $\text{M}$ with a numerical method. We numerically calculate the value of ${\mathbf{p}}$ by running a simulation of the system for about $10^9$ years until it reaches a state where the coordinates only change on the order of $10^{-9}$ after one oscillation. We then introduce a perturbation ${\boldsymbol{\delta }}$ to either A or P, and simulate the system to find the resulting coordinates ${\mathbf{p}} + {\boldsymbol{\delta }}'$ after 40 kyr.

6a $$\begin{aligned}\text{M}\left( {\mathbf{p}} + {\boldsymbol{\delta }} - {\mathbf{p}}\right) \approx {\mathbf{p}} + {\boldsymbol{\delta }}' - {\mathbf{p}} \end{aligned}$$

6b $$\begin{aligned}\text{M}{\boldsymbol{\delta }} \approx {\boldsymbol{\delta }}'. \end{aligned}$$

Let ${\boldsymbol{\delta}}$ be either the column vector $\delta [1;0]$ to calculate the right column of $\text{M}$ or $\delta [0;1]$ to calculate the left column. The magnitude of perturbation $\delta$ was found to give the most accurate calculations for values between the orders of $10^{-3}$ and $10^{-6}.$

The eigenvalues of the $2\times 2$ matrix $\text{M}$ are the Floquet multipliers and inform us of the rate at which the system converges to the fixed point. One multiplier is generally equal to unity; the corresponding eigenvector corresponds to the direction of the velocity vector for the orbit at the point ${\mathbf{p}}.$ The absolute value of the second eigenvalue indicates how much the system decays after one revolution. Letting $\lambda$ be the absolute value of the second eigenvalue, we find the time constant (how long for the amplitude to decay by a factor of *e*) to be $-40000/\ln \left( \lambda \right) .$ Keeping with the example above, the 40 kyr in the equation is the time of one revolution.

The same technique is used to calculate the time constant for systems where the periodicity is exponentially increasing. A negative sign is introduced into the differential equation to run the simulation backwards so that the periodicity is exponentially decreasing. Explicitly, we use the equations:

7a $$\begin{aligned}\frac{dA}{dt} = -\left[ I_0\left( 1 + \gamma S\left( A\right) \right) - kA\exp \left( P \right) \right] \end{aligned}$$

7b $$\begin{aligned}\frac{dP}{dt} = -\left[ kA - M\right] . \end{aligned}$$

Note that the periodic forcing $k(t)=k(-t),$ so time reversal does not affect this term (cf. Eq. (3)).

Alternatively, the decay rate can be estimated by fitting an exponential function to the envelope of the alkalinity, as demonstrated in Fig. 9. In Fig. 7, results using this approach are compared with Floquet calculations. The slightly different values from the two methods may be the result of several factors. Firstly, the value found by fitting is sensitive to which points are included in the fit. Secondly, the Floquet analysis relies on a linearization and the actual decay rate may be affected by higher-order terms that are not taken into account. Thirdly, there is sensitivity to the magnitude of perturbation in Floquet analysis. If the perturbation is too small, numerical noise starts to have a major impact. If the perturbation is too large, the Floquet theory does not provide a good approximation due to higher-order effects. Thus, one needs to find a balance with regard to the perturbation magnitude. Even so, note that consistent values are found across several orders of magnitude (Fig. 7).

## Appendix 3: Impact of forcing period

An important question considered over many years (Hays et al. 1976; Imbrie et al. 1993; Huybers and Wunsch 2005; Huybers 2011) is whether obliquity or precession is the dominant forcing of the glacial–interglacial cycles. In this paper, we focused primarily on obliquity, which has a 40-kyr period, as the forcing mechanism. In this Appendix, we consider the effect of instead using a 20-kyr period due to precession as the forcing.

Simulations identical to the jump in feedback strength performed earlier in the paper are repeated with the periodic forcing set to a period of 20 kyr (Fig. 1). The response time for the shift from $\gamma = 0.09$ to $\gamma = 0.12$ is $\sim 10$ My, much longer than in the earlier simulation (Fig. 6c/6d). This is because the current simulation converged to a stable fixed periodicity for $\gamma = 0.09,$ rather than the oscillating periodicity seen in Fig. 6c/6d. However, in general, the time scale of the response is not seen to have any consistent increase or decrease and is roughly the same as with the 40-kyr forcing (Fig. 10).

## Footnotes

1 https://github.com/GlacialCycles/Example-Code.

2 https://docs.scipy.org/doc/scipy/reference/generated/scipy.signal.find_peaks.html.
